# Prosthetic Design Factors Influencing Peri-Implant Disease: A Comprehensive Review

**DOI:** 10.7759/cureus.48737

**Published:** 2023-11-13

**Authors:** Pallavi Chankhore, Sheetal R Khubchandani, Amit Reche, Priyanka Paul

**Affiliations:** 1 Public Health Dentistry, Sharad Pawar Dental College and Hospital, Datta Meghe Institute of Higher Education and Research, Wardha, IND; 2 Prosthodontist and Implantologist, Sharad Pawar Dental College and Hospital, Datta Meghe Institute of Higher Education and Research, Wardha, IND

**Keywords:** interdisciplinary collaboration, soft tissue health, mechanical stress, microbial colonization, prosthetic design, peri-implant disease

## Abstract

Peri-implant disease, encompassing peri-implant mucositis and peri-implantitis, presents a growing challenge in implant dentistry. This comprehensive review explores the intricate interplay between prosthetic design factors and the development of peri-implant disease. By analyzing the impact of prosthetic components on microbial colonization, mechanical stress, and soft tissue health, the review highlights their crucial role in disease prevention and management. Additionally, it emphasizes the significance of maintenance protocols, prosthetic adjustments, and patient education in ensuring favorable long-term outcomes. The review underscores the potential for future advancements in prosthetic design, including innovative materials and digital technologies, and stresses the importance of interdisciplinary collaboration in optimizing patient care. Overall, the review underscores the critical role of prosthetic design in addressing the complexities of peri-implant disease, offering insights for clinicians and researchers to enhance the success and longevity of implant-supported restorations.

## Introduction and background

Peri-implant disease refers to inflammatory conditions affecting the tissues surrounding dental implants, leading to the loss of supporting bone and potential implant failure. It encompasses two main categories: peri-implant mucositis, characterized by inflammation confined to the soft tissues, and peri-implantitis, involving both soft tissue inflammation and progressive bone loss around the implant [1].

Comprehending the intricate relationship between prosthetic design factors and peri-implant disease is crucial for achieving long-term success in implant dentistry. Prosthetic components play a significant role in influencing the biological response of peri-implant tissues, affecting the stability and longevity of dental implants. Therefore, a thorough grasp of how various design elements interact with the surrounding oral environment is imperative for improving treatment outcomes and minimizing peri-implant complications [2].

This comprehensive review aims to critically evaluate the existing literature on the impact of prosthetic design factors on peri-implant disease. By analyzing and synthesizing current research findings, this review seeks to provide a comprehensive understanding of the intricate relationship between prosthetic design and peri-implant health. Additionally, this review will outline potential implications for clinical practice and offer insights into future research directions, ultimately contributing to improving treatment protocols and the long-term success of dental implant therapy.

## Review

Overview of peri-implant disease

Types of Peri-Implant Disease

Peri-implant mucositis: Peri-implant mucositis is an early-stage peri-implant disease characterized by inflammation restricted to the soft tissues surrounding dental implants. This condition often presents visible clinical signs, including redness, swelling, and bleeding when probing the gums. While it is not as severe as peri-implantitis, peri-implant mucositis is a significant concern as it is a precursor to the more advanced form of the disease. Fortunately, peri-implant mucositis can frequently be managed with appropriate intervention. This intervention primarily involves professional maintenance procedures performed by dental professionals, such as scaling and root planning around the affected implant and improved oral hygiene practices advised to the patient. Timely and effectively managing peri-implant mucositis is critical in preventing its progression into peri-implantitis, a more challenging condition to treat [3].

Peri-implantitis: Peri-implantitis represents a more severe and advanced stage of peri-implant disease. It extends beyond the soft tissues, affecting the supporting bone around the dental implant. As peri-implantitis progresses, there is a notable loss of bone structure, which can ultimately lead to implant failure if left untreated. This condition poses a substantial challenge in implant dentistry, as its management typically requires more complex and aggressive interventions. Dental professionals may need surgical debridement to remove infected tissue and deposits around the implant. Additionally, antimicrobial therapy may combat bacterial infection contributing to the disease. In severe cases, when bone loss is extensive and the implant is compromised, removal of the implant might be the only viable solution. The management of peri-implantitis is not only intricate but also highlights the importance of early detection and effective preventive measures to avoid its development [4].

Prevalence and Incidence Rates

The prevalence and incidence rates of peri-implant disease vary across studies and populations. Nevertheless, it is a growing concern in implant dentistry as more individuals opt for dental implants to replace missing teeth. The prevalence of peri-implant mucositis has been reported to range from 19% to 65% of implant sites. In comparison, the prevalence of peri-implantitis is estimated to affect 9% to 56% of patients with dental implants [5]. Various factors, including patient-related characteristics, such as oral hygiene practices and systemic health, and implant-related factors, such as prosthetic design, influence incidence rates. Understanding the prevalence and incidence of peri-implant disease is essential in recognizing its public health impact and the need for effective prevention and management strategies [6].

Etiology and Risk Factors

Poor oral hygiene: Inadequate oral hygiene practices present a primary risk factor for peri-implant disease. When patients fail to control plaque buildup around dental implants effectively, it creates an environment conducive to the accumulation of harmful bacteria and the formation of biofilms. The initiation of the inflammatory process is a direct result of these microbial activities. Proper oral hygiene, including regular and thorough cleaning of implant-supported restorations, is essential to prevent peri-implant disease [7].

Smoking: Smoking is a well-established and significant risk factor for the development of peri-implant disease. This habit has been found to impair the body's immune response and reduce blood flow to the tissues surrounding the implant. Consequently, smokers are more susceptible to infections and inflammation, making them at higher risk for peri-implant complications. Smoking cessation and counseling should be a crucial part of the management and prevention of peri-implant disease in this patient population [8].

Systemic health conditions: Individuals with certain systemic diseases, such as diabetes and immunosuppressive conditions, face an elevated risk of peri-implant complications. These underlying health issues can compromise the body's ability to mount an effective immune response, making it less capable of defending against bacterial threats. Dental professionals must exercise caution and closely monitor patients with systemic health conditions, emphasizing the importance of meticulous oral hygiene and timely intervention to reduce the risk of peri-implant disease [9].

Prosthetic factors: Prosthetic design elements, including the type of crown and abutment used, can have a notable influence on the risk of peri-implant disease. The choice of prosthetic components, materials, and contours can affect microbial colonization, mechanical stress, and soft tissue health. Understanding how these prosthetic design factors interact with the oral environment is vital in making informed decisions to reduce the risk of peri-implant complications. Prosthodontists and implantologists are pivotal in selecting appropriate prosthetic components to mitigate these risks and improve long-term implant success [10].

Prosthetic design factors

Prosthetic design factors encompass a range of considerations related to the fabrication and configuration of dental prostheses, particularly those supported by dental implants. These factors include the selection of materials, the design of crowns and abutments, and the overall prosthesis geometry. They play a pivotal role in determining the functional and esthetic success of implant restoration, but they also have a significant influence on peri-implant health [11].

Role of Prosthetic Components in Peri-Implant Health

Prosthetic components are paramount in peri-implant health, primarily due to their intimate interactions with the surrounding oral environment. The materials and design decisions made during the prosthetic phase of implant dentistry can exert profound effects on the long-term stability of the implant and the health of the peri-implant tissues. Understanding these intricate interactions is a fundamental cornerstone for achieving optimal outcomes in implant dentistry and effectively mitigating the risk of peri-implant disease [2].

The choice of materials for prosthetic components holds considerable weight in ensuring peri-implant health. Biocompatible materials resistant to corrosion and wear are pivotal for minimizing adverse reactions and tissue irritation, which can contribute to peri-implant complications. The selection of materials for crowns, abutments, and other components must be guided by an understanding of their compatibility with the patient's oral environment and systemic health [12].

Furthermore, the design of prosthetics, such as the emergence profile, contours, and occlusal scheme, profoundly influences peri-implant health. These design choices can determine the ease with which patients can maintain proper oral hygiene around the implant-supported prosthesis. Poorly designed prosthetics can create areas prone to food impaction and bacterial colonization, making patients more susceptible to peri-implant mucositis and, subsequently, peri-implantitis [13].

Overview of various prosthetic designs

Crown Design and Its Impact on Peri-Implant Health

Occlusal forces play a pivotal role in the success of dental implant procedures. The distribution of occlusal forces on the crown is a critical factor to consider. When the crown is poorly designed and does not distribute forces evenly, it can lead to excessive stress concentrations at specific points in the implant-bone interface. These localized forces can potentially damage the surrounding bone and soft tissues, increasing the risk of peri-implant complications. One important strategy to mitigate these force-related issues is platform switching. Platform switching involves using an abutment with a smaller diameter than the implant fixture. This configuration creates a horizontal mismatch between the implant and the abutment, which can help redistribute occlusal forces more favorably. A well-designed crown can further ensure that occlusal forces are distributed evenly across the implant site by incorporating platform switching, significantly reducing the risk of force-related complications [14]. Implementing platform switching is an important consideration in the overall success of dental implant treatments.

Emergence profile: The emergence profile, which is how the crown emerges from the soft tissue, is another vital element in crown design. An optimal emergence profile should promote healthy soft tissue contours and facilitate easy access for oral hygiene. Inadequate emergence profiles may create challenges in maintaining proper oral hygiene, as it can lead to food impaction areas and bacterial colonization. The subsequent inflammation and infection contribute to developing peri-implant mucositis and, ultimately, peri-implantitis [15].

Crown-to-implant ratio: The proportion of the crown to the implant fixture, known as the crown-to-implant ratio, plays a role in peri-implant health. Imbalances in this ratio, particularly in cases with excessive crown height, can result in an uneven distribution of forces. This can lead to mechanical complications and increased strain on the implant-bone interface, which may accelerate bone loss and peri-implant disease [16].

Abutment Design and Its Influence on Peri-Implant Disease

Emergence profile: The emergence profile created by the abutment design plays a pivotal role in determining peri-implant health. This profile defines how implant-supported restoration emerges from the soft tissues, and it has both functional and esthetic implications. An optimal emergence profile supports the formation of healthy and natural soft tissue contours around the implant. This not only enhances the esthetic appearance of the restoration but also aids in maintaining the health of the peri-implant soft tissues. In contrast, poorly designed abutments can result in irregular or unfavorable emergence profiles. These irregularities can create challenges in oral hygiene maintenance as they may lead to food impaction areas and the accumulation of bacterial biofilm. This, in turn, can contribute to the development of peri-implant mucositis, an early stage of peri-implant disease. Irregular emergence profiles are more likely to trap debris and bacteria, making them a risk factor for soft tissue complications [17].

Soft tissue contours: The design of the abutment has a direct impact on the contours of the surrounding soft tissues. Ideally, abutments should encourage the formation of well-adapted and healthy soft tissue around the implant. When abutment design fails to support these favorable soft tissue contours, it can result in soft tissue recession. This recession creates pockets or crevices in the peri-implant area, where bacteria can readily accumulate. Bacterial biofilm within these recesses increases the risk of inflammation and infection, which is a characteristic of peri-implant disease. Therefore, an abutment design that promotes proper soft tissue contours is essential for maintaining peri-implant health [18].

Accessibility for oral hygiene: Effective oral hygiene maintenance is a cornerstone of peri-implant disease prevention. Poorly designed abutments can impede the patient's ability to clean around the implant-supported prosthesis effectively. There are several reasons for this. Irregular abutment contours can create challenges in accessing certain areas around the implant, making it difficult for the patient to reach and clean these spots. Additionally, the presence of undercuts or areas that are challenging to reach with dental instruments can trap debris, making proper cleaning even more problematic. When patients struggle to maintain proper oral hygiene due to these design-related issues, the risk of peri-implant mucositis and peri-implantitis significantly increases. Inadequate cleaning can result in the accumulation of microbial biofilm, inflammation, and the progression of peri-implant disease [19].

Prosthesis-Material Interactions and Their Implications

Biocompatibility: Biocompatibility is a paramount consideration in selecting materials for prosthetic components. Biocompatible materials are those the body tolerates well without causing adverse reactions or inflammatory responses. When biocompatible materials are used, the risk of complications such as localized tissue irritations, foreign body reactions, and implant failure is significantly reduced. A deep understanding of the biocompatibility of materials is essential to prevent these complications and promote overall peri-implant health. Biocompatible materials are especially crucial in implant dentistry, as they come into direct contact with oral tissues for extended periods [20].

Corrosion resistance: The oral environment is exceptionally demanding, exposed to variations in pH, temperature, and chemical agents. Materials selected for prosthetic components must resist corrosion and degradation over time. Corrosion can release ions and particles into the surrounding tissues, potentially triggering inflammation and adverse reactions. The choice of corrosion-resistant materials is vital to ensure the long-term stability and health of the peri-implant tissues. Using materials that can withstand the challenges of the oral environment helps minimize the risk of corrosion-related complications [21].

Esthetic considerations: Esthetics play a significant role, particularly in the anterior region of the mouth, where implant-supported prostheses must blend seamlessly with the natural dentition. Esthetic materials contribute to patient satisfaction and confidence, enhancing the overall quality of life. However, it is crucial to balance esthetics and material properties. Esthetic considerations should not come at the expense of biocompatibility and durability. Finding materials that offer pleasing esthetics and compatibility with oral tissues is essential for patient satisfaction and the long-term health of peri-implant tissues. The right balance ensures that the implant-supported restoration looks natural, maintains its structural integrity, and supports the surrounding tissues effectively [22].

Influence of prosthetic design factors on peri-implant disease

Microbial Colonization and Biofilm Formation About Prosthetic Design

The role of microbial colonization and biofilm formation in peri-implant disease is a critical aspect of implant dentistry. Prosthetic design plays a substantial role in determining the extent to which harmful microorganisms can adhere to implant surfaces and prosthetic components, and these interactions are pivotal in understanding and preventing peri-implant disease [23].

Surface roughness: The surface roughness of prosthetic materials and components is a crucial factor influencing microbial colonization. Irregular and rough surfaces provide niches for bacteria to attach and form biofilms. Prosthetic materials with a smoother surface reduce the opportunities for bacterial attachment, making it more challenging for biofilms to develop. The design and manufacturing of prosthetic components must aim to minimize surface roughness, ultimately hindering microbial colonization and reducing the risk of peri-implant disease [24].

Emergence profile: The emergence profile created by prosthetic components, such as abutments, significantly affects microbial colonization. An optimal emergence profile should promote a seamless transition from the implant to the prosthetic crown while minimizing areas where bacteria can accumulate. Poorly designed emergence profiles can create crevices and pockets that provide shelter for bacteria, facilitating biofilm formation. Biofilms can induce inflammation and peri-implant mucositis, which, if uncontrolled, may progress to peri-implantitis [25].

Mechanical Stress and Its Effect on Peri-Implant Tissues

Mechanical stress, arising from the occlusal forces experienced during biting and chewing, is a significant determinant in the development of peri-implant disease. The design of the prosthesis and the occlusal scheme employed substantially impact how these forces are distributed across the implant and the surrounding bone. Understanding the implications of mechanical stress on peri-implant tissues is essential for preserving long-term peri-implant health [26].

Distribution of occlusal forces: The distribution of occlusal forces across the implant and surrounding bone is critical in preventing peri-implant disease. When the prosthesis is inadequately designed or the occlusal scheme is unbalanced, there is a risk of creating localized stress concentrations. These areas of concentrated forces can result in excessive mechanical stress on the implant-bone interface, which may lead to bone loss over time. Proper prosthetic design must consider the even distribution of occlusal forces to reduce the risk of mechanical complications [27].

Prosthetic design factors significantly influence the management and distribution of occlusal forces in implant-supported restorations. These factors encompass the design of the crown, the implant's angulation, and the choice of materials. In cement-retained prostheses, the crown's design plays a critical role, as it must be carefully engineered to ensure it can withstand and evenly distribute occlusal forces. Additionally, the angulation of the implant is vital to align with the patient's natural occlusion, preventing any misdirected forces. The choice of materials also becomes pivotal, as it should consider their strength and their ability to withstand mechanical stress. For screw-retained prostheses, similar principles apply. The crown's design is paramount to effectively managing occlusal forces. In this case, the choice of materials may include factors related to the screw and the abutment's design. The angulation of the implant should once again align with the patient's natural occlusion, mitigating the risk of misdirected forces. A comprehensive understanding of how these factors interplay is essential for minimizing the risk of bone loss and implant failure [28].

Prosthetic Contours and Their Impact on Soft Tissue Health

Food impaction and oral hygiene: Poorly contoured prosthetic elements can create spaces and crevices where food particles can become trapped, leading to food impaction. This impaction can be challenging for patients to manage during oral hygiene routines, as standard toothbrushes and floss may not adequately clean these areas. The accumulation of debris and the difficulty in maintaining proper oral hygiene promote the growth of bacterial biofilms, ultimately contributing to the development of peri-implant mucositis [29].

Emergence profile: The emergence profile, the way the prosthetic component emerges from the soft tissue, is a critical factor influencing soft tissue health. An optimal emergence profile promotes natural-looking and healthy soft tissue contours, ensuring that the soft tissues hug the implant and prosthetic components. Poorly designed emergence profiles can lead to unsightly black triangles, soft tissue recession, and inadequate papilla preservation, negatively affecting esthetics and oral hygiene. Such unfavorable contours can make the patient susceptible to peri-implant complications [30].

Soft tissue thickness: The thickness of the soft tissue around the implant site is also influenced by prosthetic contours. Properly designed contours help maintain adequate soft tissue thickness, providing better support for the papilla and minimizing the risk of recession. Inadequate soft tissue thickness can make the implant site more vulnerable to bacterial infiltration and peri-implant mucositis [31].

Management and prevention strategies

Role of Maintenance Protocols in Preventing Peri-Implant Disease

Regular follow-up appointments are the linchpin of effective maintenance protocols for implant-supported prostheses. They serve as a proactive mechanism for monitoring and safeguarding peri-implant health. During routine follow-up appointments, dental professionals meticulously assess the health of peri-implant tissues, allowing them to detect any subtle changes or signs of trouble early on. This includes monitoring the implant's stability and tracking any deviations from normal function or mobility. Early detection at this stage is crucial because it allows timely intervention, potentially preventing the progression of peri-implant mucositis to more severe peri-implantitis. By identifying issues at their inception, clinicians can implement appropriate treatment strategies and provide the necessary guidance to mitigate complications, preserving the longevity and functionality of the implant-supported prosthesis [32].

Professional cleanings: The significance of professional cleanings in maintenance protocols cannot be overstated. These clinical interventions, which include scaling and root planning, are instrumental in maintaining the cleanliness and health of implant surfaces and prosthetic components. They serve to remove microbial biofilm and calculus deposits that may accumulate over time. Routine oral hygiene practices at home do not effectively remove such deposits, making professional cleanings essential for maintaining peri-implant health. By ensuring that the implant surface and surrounding tissues remain free from harmful bacterial accumulations, professional cleanings contribute significantly to the prevention of peri-implant disease. Their role extends beyond hygiene, as they help to create a favorable environment for implant health and contribute to the long-term success of the restoration [33].

Assessments of peri-implant health: Routine assessments of peri-implant health are a comprehensive diagnostic approach employed during maintenance protocols. This involves a thorough clinical examination, often complemented by radiographic evaluations. These assessments are integral to identifying changes in the health of soft tissues, any alterations in bone levels, and early signs of inflammation or infection. Dental professionals often use probing to measure pocket depths around implants, providing valuable diagnostic information. Through these assessments, dental providers can detect peri-implant complications at an early stage, even before clinical symptoms manifest. This early detection is pivotal, as it enables timely intervention to address issues and prevent their escalation into more severe and complex conditions. By regularly assessing peri-implant health, clinicians are equipped to make informed decisions and provide timely care, ultimately preserving the health and function of the implant-supported prosthesis [34].

Patient education: Patient education is a cornerstone of maintenance protocols that empower individuals with implant-supported prostheses to participate actively in oral health. Educating patients about the significance of maintaining proper oral hygiene practices is paramount. Patients must know the importance of adhering to recommended recall schedules and recognizing the early signs of potential peri-implant disease. An informed patient is likelier to take ownership of their oral health care, diligently follow through with maintenance appointments, and promptly communicate any concerns or changes they notice. This patient-provider collaboration is invaluable in achieving and maintaining peri-implant health [35].

Evidence-based guidelines: Developing and implementing evidence-based maintenance guidelines are a foundation for delivering consistent and effective peri-implant care. These guidelines provide dental professionals with a structured framework for patient management, ensuring that best practices are consistently followed. They incorporate the latest research findings and clinical evidence, keeping dental providers up-to-date with the ever-evolving field of implant dentistry. Evidence-based guidelines facilitate standardized care, ensuring that all patients with implant-supported prostheses receive a high level of care that aligns with the best available knowledge. These guidelines are essential in promoting the long-term health and success of implant-supported restorations, reducing the risk of peri-implant disease [36].

Implications for Prosthetic Modifications and Adjustments

Prosthetic component modifications: Prosthetic component modifications represent a proactive approach to managing peri-implant disease. These adjustments involve a meticulous assessment of the contours and materials of crowns and abutments, aiming to optimize oral hygiene access and reduce the risk of bacterial colonization. The process often entails reshaping or redesigning components to eliminate potential areas prone to food impaction or the accumulation of microbial biofilms. By creating smooth and well-contoured prosthetic components, dental professionals aim to minimize the retention of debris and facilitate effective self-care practices by the patient. These modifications contribute to the overall maintenance of peri-implant health and prevent the onset or progression of peri-implant disease, fostering a favorable environment for the long-term stability of the implant-supported restoration [37].

Minimizing mechanical stress: Prosthetic adjustments directed at minimizing mechanical stress are pivotal in ensuring the biomechanical equilibrium of the implant-bone interface. When unbalanced occlusal forces are identified as potential contributors to peri-implant disease, adjustments to the occlusion become imperative. These adjustments are designed to achieve a more uniform distribution of forces, reducing the risk of further tissue damage. By reconfiguring the occlusal scheme, dental professionals can create a harmonious balance that alleviates undue pressure on the implant and surrounding tissues. The objective is to establish biomechanically favorable conditions, safeguarding the implant's structural integrity and minimizing the likelihood of complications that may compromise peri-implant health [37].

Integrated therapeutic approach: Prosthetic modifications and adjustments are most effective when integrated into a comprehensive therapeutic approach to peri-implant disease. This integrated framework acknowledges the multifactorial nature of peri-implant health and addresses the condition from various angles. Alongside prosthetic modifications, this approach encompasses other therapeutic measures, including professional cleanings, antimicrobial therapies, and patient education. By combining these interventions, dental professionals can develop a cohesive strategy that controls the progression of peri-implant disease and mitigates its detrimental impact on peri-implant health. This comprehensive approach emphasizes the importance of holistic care, emphasizing the need for multidimensional interventions to ensure the long-term success and stability of the implant-supported prosthesis [10].

Strategies for Patient Education and Long-Term Oral Hygiene Maintenance

Patient education: Education is the foundation for preventing peri-implant disease. Dental professionals should impart essential information to patients regarding proper oral hygiene practices. This includes educating patients on the importance of effective cleaning around the implant-supported prosthesis, which may require unique cleaning. Patients need to understand that their active participation in oral care is critical for the long-term success of their implants. Furthermore, patients should be informed about the risk factors associated with peri-implant disease and be aware of the potential signs, such as bleeding gums or discomfort. Equipping patients with this knowledge empowers them to recognize early warning signs and take timely action. Additionally, emphasizing the necessity of regular dental check-ups and professional cleanings is essential. This regular follow-up ensures that peri-implant health is monitored and professionally maintained [38].

Tailored oral hygiene strategies: Patient education goes hand in hand with developing personalized long-term oral hygiene maintenance strategies. These strategies should be tailored to each patient's specific needs and circumstances. Notably, the patient's abilities, preferences, and any existing conditions should be considered. For example, patients with dexterity limitations may benefit from specialized oral hygiene aids, such as interdental brushes, adaptive flossing devices, or water flossers. These tools help facilitate effective cleaning around implant-supported prostheses, ensuring that patients maintain proper oral hygiene despite potential challenges [39].

Oral hygiene plans: A personalized oral hygiene plan is essential for preserving peri-implant health. The plan should consider the patient's unique needs and oral health status. It may involve a schedule for regular check-ups and professional cleanings, considering any additional measures required, such as antimicrobial rinses or prescription-strength toothpaste. Patients should be actively engaged in developing their oral hygiene plans, ensuring that the strategies align with their abilities and preferences. A well-structured and tailored plan reinforces the patient's commitment to oral health and encourages proactive participation in maintaining peri-implant health [40].

Future directions and concluding remarks

Potential Advancements in Prosthetic Design to Mitigate Peri-Implant Disease

Advanced prosthetic materials: One of the most promising avenues for reducing the risk of peri-implant disease lies in developing advanced prosthetic materials. Research in materials science is continually pushing the boundaries of biocompatibility and corrosion resistance. These advancements may create materials that are even more harmonious with the oral environment, minimizing the risk of adverse reactions or sensitivities. Enhanced material properties can also contribute to the longevity of implant restorations, reducing the need for replacements or revisions. The integration of novel materials can lead to implant-supported prostheses that are not only more robust but also better tolerated by the host tissues [41].

Digital technologies and precision prosthetics: The integration of digital technologies, such as CAD/CAM (computer-aided design/computer-aided manufacturing), is transforming the field of prosthetic design. These technologies enable the creation of exact and patient-specific prosthetic components. Through advanced imaging and computer modeling, clinicians can design restorations that optimize the fit, function, and esthetics of implant-supported prostheses. The result is a more tailored and individualized approach to prosthetic design, enhancing patient comfort and satisfaction. Precise prosthetic fit can help minimize gaps and crevices that might otherwise contribute to food impaction and bacterial colonization, reducing the risk of peri-implant disease [42].

Evaluation of emerging prosthetic solutions: Understanding how emerging prosthetic materials and digital technologies influence peri-implant health is pivotal in advancing the field. Ongoing research and clinical studies are essential to evaluate the safety and efficacy of these innovative solutions. In-depth assessments can help identify any potential risks or benefits associated with new materials and design approaches. This information guides clinicians in deciding which prosthetic options are most suitable for their patients [43].

Importance of Interdisciplinary Collaboration for Improved Patient Outcomes

A multidisciplinary team of dental specialists: In implant dentistry, an interdisciplinary team is often comprised of periodontists, prosthodontists, oral surgeons, and dental hygienists, each with their unique expertise. Collaborative efforts among these specialists ensure that the patient's needs are addressed comprehensively. Periodontists, for instance, specialize in the health of the supporting structures, prosthodontists focus on the prosthetic aspects, and oral surgeons manage the surgical phases of implant therapy. Dental hygienists are crucial in maintaining oral health, including peri-implant tissues. This collaboration results in more efficient treatment planning and execution, with each specialist contributing their knowledge and skills to achieve optimal patient outcomes [44].

Long-term care and maintenance: Interdisciplinary collaboration extends beyond the initial phases of implant treatment. It is equally essential during long-term maintenance, where dental hygienists and other specialists work together to ensure that the patient's oral health and implant function are preserved. Regular assessments, professional cleanings, and any necessary prosthetic adjustments are managed cohesively. This ongoing collaboration plays a crucial role in preventing peri-implant disease and enhancing the longevity of implant-supported restorations [45].

Communication with medical professionals: Collaboration with medical professionals is especially vital when patients have systemic health issues. Many systemic conditions, such as diabetes or immunosuppressive disorders, can influence the outcome of implant therapy. By working closely with physicians and specialists outside of dentistry, dental professionals can coordinate care, address potential complications, and optimize treatment plans. This holistic approach ensures that the patient's overall health is considered and that any specific medical considerations are incorporated into the implant treatment plan [46].

Patient-centered approach: Interdisciplinary collaboration places the patient at the center of care. It acknowledges that each patient is unique and may have specific medical or dental needs. The collective expertise of an interdisciplinary team allows for a tailored approach that aligns with the patient's circumstances, resulting in treatments that are not only clinically effective but also patient-centered [47].

## Conclusions

In conclusion, the relationship between prosthetic design and peri-implant disease is a critical factor in implant dentistry. Prosthetic elements, including crowns, abutments, and materials, play a central role in determining the health and longevity of implant-supported restorations. This comprehensive review has highlighted critical insights into the significance of prosthetic design in managing peri-implant disease. It has emphasized how design factors impact microbial colonization, mechanical stress, and soft tissue health, all critical considerations in preventing peri-implant complications. Additionally, the review has underlined the importance of maintenance protocols, prosthetic adjustments, and patient education as vital components of a holistic approach to peri-implant disease management. Looking forward, ongoing advancements in materials and digital technologies offer promising prospects for improving peri-implant health. Furthermore, the collaborative efforts of interdisciplinary healthcare professionals will continue to enhance patient outcomes by considering both oral and systemic health factors. In sum, prosthetic design is a fundamental determinant in the management of peri-implant disease, shaping the success and overall well-being of implant patients.
